# Figure of Merit for Gas Overtone Spectroscopy on a Chip in Near-Infrared (NIR)

**DOI:** 10.3390/s25165092

**Published:** 2025-08-16

**Authors:** Uzziel Sheintop, Alina Karabchevsky

**Affiliations:** School of Electrical and Computer Engineering, Ben-Gurion University of the Negev, Beer-Sheva 8410501, Israel

**Keywords:** gas sensing, overtone spectroscopy, silicon photonics, near-infrared, on-chip sensors

## Abstract

The development of compact, CMOS-compatible gas sensors is critical for advancing real-time environmental monitoring and industrial diagnostics. In this study, we present a detailed numerical investigation of integrated photonic waveguide designs—such as ridge and slot—optimized for overtone-based gas spectroscopy in the near-infrared range. By evaluating both the evanescent-field confinement and curvature-induced losses across multiple silicon-on-insulator platforms, we identify optimal geometries that maximize light–analyte interactions while minimizing bending attenuation. Our findings provide essential design guidelines for high-performance, low-footprint gas sensors.

## 1. Introduction

The demand for compact, highly sensitive, and cost-effective gas sensors has surged across fields ranging from environmental monitoring and industrial process control to medical diagnostics and homeland security. Integrated photonic platforms—especially those based on silicon-on-insulator (SOI) technology—offer an attractive path toward miniaturized gas detection systems that combine scalability with robust performance [1]. In particular, near-infrared (NIR) overtone spectroscopy has emerged as a powerful technique due to its compatibility with mature telecommunication components, low optical losses, and the ability to perform real-time, label-free analysis.

Recent reviews have underscored the versatility of guided-wave photonic sensors across the infrared spectrum, highlighting near-infrared solutions for their compactness, low propagation loss, and compatibility with telecom-grade components [2,3,4]. In addition, overtone spectroscopy in the NIR has been recognized as a promising approach for enabling compact and CMOS-compatible gas sensors [5]. However, designing waveguide architectures that simultaneously optimize light–analyte interactions and minimize bending-induced losses remains a key challenge. Here, we present a systematic numerical study to address this challenge, offering actionable design principles for next-generation photonic gas sensors.

Guided wave structures allow the manipulation of light in compact dimensions for various purposes. They can be used for remote and on-site detection, such as bio-sensing [2].

The fraction of the power that takes part in the interaction with the analyte in the vicinity of the waveguide is known as Γ, also called the evanescent-field ratio (EFR) or confinement factor.

Besides manipulating and enhancing the fraction of the power that takes part in the interaction with the analyte (Γ), it is also important to have as large an interaction length as possible. This is according to the Beer–Lambert law. In the limited area of a standard chip, this can be achieved by the curved design of the on-chip sensor [6].

Many studies were performed to design and characterize waveguides for gas sensing. These studies include a comprehensive numerical investigation of the waveguides, aiming to optimize confinement factors and losses for different architectures, dimensions, and materials. The fraction of light that interacts with the target analyte depends on the dimensions, substrate, and guiding material of the waveguide, as well as the wavelength. Extensive numerical studies were performed in the mid-infrared (MIR) segment for different waveguide architectures to demonstrate gas sensors based on the evanescent field, among them: rib [3,7,8], ridge [6], strip [9], and slot [10,11,12].

Compared to the MIR, in the near-infrared (NIR) region, there are fewer works conducted. Besides practical works [6,13,14,15], there were few numerical studies in the NIR [16,17]. Previous studies have demonstrated enhanced NIR gas sensing performance using photonic crystal slot waveguides and subwavelength grating (SWG) structures, enabling slow-light effects and sub-ppm methane detection [13,14,18]. Kita et al. [19] further compared strip, slot, and SWG waveguides, highlighting their high confinement factors but also noting increased fabrication sensitivity and propagation losses. Additional numerical investigations have modeled slot and SWG waveguides under NIR conditions, showing potential for broadband spectral overlap and multi-gas sensing [16,20,21]. Despite these advances, a comprehensive numerical comparison of ridge and slot architectures under standard SOI constraints—jointly addressing confinement and bending loss—has remained underexplored. Our work aims to fill this gap.

This gap largely stems from the relatively weaker light absorption of NIR overtones compared to MIR fundamentals, as well as higher scattering losses due to increased sensitivity to surface roughness at shorter wavelengths. According to the Rayleigh criterion, these scattering losses become more pronounced in the NIR as the shorter wavelengths are more sensitive to surface roughness, leading to greater light redirection and attenuation. The weaker absorption of overtones arises due to the smaller transition dipole moments and lower probabilities of these higher-order transitions, as dictated by the anharmonicity of molecular vibrations. This anharmonicity becomes increasingly significant at higher-order transitions, further reducing the overlap of vibrational wavefunctions and weakening the absorption.

Unlike MIR devices, where high absorption allows for compact straight waveguides, NIR systems rely on long interaction lengths, often requiring curved layouts that introduce additional attenuation. Moreover, the shorter NIR wavelengths increase susceptibility to scattering losses from fabrication-induced surface roughness, exacerbating total propagation loss. These wavelength-dependent challenges, often underappreciated in earlier NIR modeling works, motivate the need for a comprehensive analysis of performance trade-offs under practical constraints. Our work responds to these NIR-specific limitations by jointly analyzing the confinement factor and bending-induced attenuation across different waveguide geometries. This dual optimization provides a practical framework for guiding design decisions within standard SOI platforms.

However, employing the NIR for sensing presents substantial benefits, including using mature sensor and light source technologies [4]. Moreover, NIR systems offer lower costs and simplicity, better signal-to-noise ratios, enhanced environmental stability, and faster data acquisition rates, making them an attractive option for various sensing applications. A comprehensive investigation of NIR technologies could reveal significant potential despite being less studied.

While achieving an optimal waveguide design is essential for maximizing gas sensing capabilities, it is also crucial to address the issue of bending losses, which can critically affect both performance and practicality.

In waveguide-based sensing applications, accurate understanding and control of bending losses are very important. Gas sensors that rely on integrated photonic waveguides often employ curved geometries to achieve compact designs or to guide light through specific sensing regions. These bends, however, introduce optical losses that can significantly reduce the signal-to-noise ratio and thus degrade the overall sensitivity and detection limits of the sensing system. By thoroughly characterizing bending losses, designers can optimize the curvature and geometry of the waveguide, ensuring more efficient light confinement and minimal unwanted attenuation.

Moreover, the careful analysis of bending losses becomes especially critical in gas sensing, where the interaction length, often dictated by the waveguide’s path, directly influences detection capabilities. In order to enhance the sensor’s performance, low bending losses must be coupled with effective gas–analyte interaction zones, which are commonly achieved through engineered waveguide layouts. This ensures high optical throughput and sufficient mode overlap with the sensing environment. A deep understanding of bending-related attenuation paves the way toward more reliable, compact, and highly sensitive integrated photonic gas sensors.

Here we report a detailed analysis of waveguide structures optimized for gas sensing in the near-infrared (NIR) range, emphasizing designs that enable longer interaction lengths while remaining compatible with accessible, cost-effective fabrication processes, and standard platforms. Additionally, we thoroughly examined and quantified bending losses under NIR operation, aiming to identify a practical balance that ensures high-performance sensing with readily implementable integrated photonic platforms.

## 2. Numerical Modeling

### 2.1. Fraction of the Power to Interact with an Analyte

Optical waveguides confine light but allow a portion of the electromagnetic field, known as the evanescent field, to extend outside the waveguide core into the surrounding medium (the analyte). The fraction of optical power in this evanescent field determines how much the light interacts with the analyte. A higher fraction of power in the analyte means stronger light–matter interactions, leading to improved sensor performance. Here, we calculated the fraction of the power propagating in each region. The fraction of the power is defined as [22]:

(1) $$\eta =\frac{P_{\;\mathrm{region}}}{P_{\;\mathrm{total}}}=\frac{{\;\int \int \;}_{\;\mathrm{region}}S\;dA}{{\;\int \int \;}_{-\infty }^{\infty }S\;dA}$$

where η is the fraction of the power, *P* is the power, *A* is the area, and S is the time-averaged Poynting vector, which is defined as [23]:

(2) $$S=\frac{1}{2}ℜ\left\{E\times H^{*}\right\}$$

where *E* is the electric field vector, *H* is the magnetic field vector, and *ℜ* is an operator that takes the real part of the product.

Using perturbation theory and based on Equations (1) and (2), one can prove that $\Gamma =f\times \frac{c/n}{v_{g}}$ [24]. Here, *f* is the filling factor that represents the fraction of the electric field residing in the analyte, *c* is the velocity of light in vacuum, and $v_{g}$ is the group velocity in a medium having effective index *n*. The explicit formulation of Γ is [3,22]:

(3) $$\Gamma =\frac{n_{g}}{n_{0}}\frac{\int \int_{\mathrm{air}}\epsilon {\left|E\right|}^{2}\;dxdy}{\int \int_{\mathrm{total}}\epsilon {\left|E\right|}^{2}\;dxdy}$$

Therefore, the high group index $n_{g}$ indicates the enhancement in the absorption of light by the analyte. Furthermore, the greater the electric field overlaps with the analyte, the greater the effective absorption by the medium is [25]. This relationship reflects the principle of the Beer–Lambert law, where the absorbance is proportional to the product of the absorption coefficient, optical path length, and the effective overlap between the mode and the absorbing medium. In the context of integrated photonic sensors, Γ acts as a multiplicative factor enhancing the effective interaction length.

The group index $n_{g}$ captures the slow-light effect, where increased dwell time of photons within the sensing region leads to enhanced absorption. This is particularly beneficial for weak overtone transitions, as it boosts the effective optical interaction per unit length. It shows that Γ can reach and even surpass 100% [3,7,26]. The group index $n_{g}$ captures the slow-light effect, where increased dwell time of photons within the sensing region leads to enhanced absorption. This is particularly beneficial for weak overtone transitions, as it boosts the effective optical interaction per unit length. As a result, Γ can reach and even surpass 100% [3,7,26].

**Figure 1:**
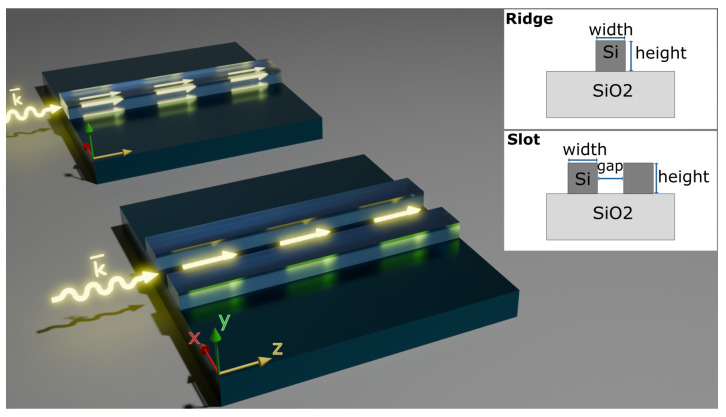
Common optical waveguide configurations used for sensing: ridge waveguide (**top**), slot waveguide (**bottom**).

**Figure 2:**
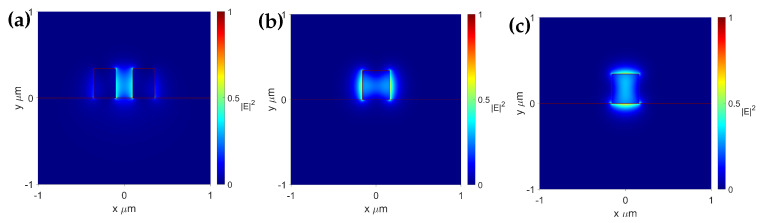
Cross -section of the E-field distribution of TE modes in (**a**) slot, (**b**) ridge (TE mode), and (**c**) ridge (TM mode) waveguides.

In our numerical calculations, we evaluated the confinement factor Γ with Lumerical MODE simulation. We examined different common configurations of waveguides used for gas sensing, including ridge and slot, as shown in Figure 1. In this study, we explored waveguide thicknesses commonly used in the waveguide fabrication industry to ensure the applicability of our research. These thicknesses were analyzed across various widths to determine the optimal configuration to achieve the highest confinement factor. Figure 2a–c shows typical E-field distributions of the TE modes in the slot and ridge waveguides, as well as the TM mode in the ridge waveguide. They illustrate the characteristic field patterns observed for the waveguides surveyed in this paper.

The confinement factor (Γ) was computed using standard simulation workflows in Lumerical MODE, with carefully selected meshing and PML boundary settings to ensure numerical stability across all geometries and wavelengths. To validate the accuracy of our results, we benchmarked our simulations against established literature values for well-characterized waveguide structures, confirming consistent trends and magnitudes.

### 2.2. Modal Behavior in Bent Waveguides

In a bent waveguide, light propagating along the curve experiences a change in its propagation conditions compared to a straight waveguide. If the bending radius is too small, a significant portion of the guided mode leaks out as radiation loss because the waveguide can no longer fully confine it. In addition to achieving a highly sensitive device, a sensing waveguide should have both a high Γ and the longest possible interaction length under the Beer–Lambert law. Within the confined area of a standard chip, this can be accomplished by using a bent waveguide pattern. However, bent waveguides exhibit losses proportional to the bending radius; therefore, it is crucial to include the analysis of the bending losses of each architecture in this study.

A key challenge in designing integrated photonic circuits is managing the bending losses that occur when waveguides are curved. One commonly used figure-of-merit for quantifying these losses is given by:

(4) $$X_{r}=\frac{\beta_{z}-\beta_{0}}{\beta_{0}}\;\cdot \;r,$$

where $\beta_{z}$ is the propagation constant of the bent waveguide, $\beta_{0}$ is the propagation constant of an equivalent straight waveguide, and *r* is the bend radius. This relationship captures how curvature modifies the local propagation properties of the guided mode. Specifically, as a waveguide is bent, the effective index profile experienced by the mode changes, leading to a shift in the propagation constant. The dimensionless ratio $\left(\beta_{z}-\beta_{0}\right)/\beta_{0}$ thus describes the relative deviation of the bent mode from its unbent counterpart, while multiplication by *r* accounts for the physical extent of the bend.

When $X_{r}$ becomes sufficiently large, the mode can no longer remain fully confined within the bent waveguide, causing radiation or scattering losses to increase. Conversely, if $X_{r}$ remains small, the waveguide curvature minimizes confinement, and the mode can propagate around the bend with relatively low loss. In practical circuit layouts, there is a constant trade-off between bend radius and device footprint: smaller radii are desirable to reduce chip size, but excessive curvature increases bending losses.

We performed a detailed analysis of the losses as a function of bending radius using a Lumerical Mode solver. According to our simulations, the investigated structures do not properly support the mode for bend radii of approximately 10–15 μm. This observation guided us to focus our study on bend radii of 20 μm and above, ensuring that the modes remain confined and that the waveguide designs are practical for real-world implementations. Furthermore, the bending loss calculations in the simulation tool leverage both a numerical overlap integral approach and the relevant Poynting-vector-based formulation of mode overlap. Specifically, the mode overlap η between two modes, defined in terms of the electric and magnetic fields, can be written as:

(5) $$\eta \;=\;\frac{{\left|\;\int \int \;\left(E_{1}\times H_{2}^{*}\right)\;\cdot \;\hat{z}\;dA\right|}^{2}}{\left(\;\int \int \;\left(E_{1}\times H_{1}^{*}\right)\;\cdot \;\hat{z}\;dA\right)\left(\;\int \int \;\left(E_{2}\times H_{2}^{*}\right)\;\cdot \;\hat{z}\;dA\right)},$$

where $E_{1}$ and $H_{1}$ are the electric and magnetic fields of mode 1, $E_{2}$, and $H_{2}$ are the corresponding fields of mode 2, and $\hat{z}$ denotes the propagation direction. This definition stems from the Poynting vector (Equation (2)) and measures how well a given mode overlaps with a reference mode in terms of power flow. Higher values of η correspond to better mode matching, indicating lower bending losses in the case of a bent waveguide compared to its unbent counterpart.

**Figure 3:**
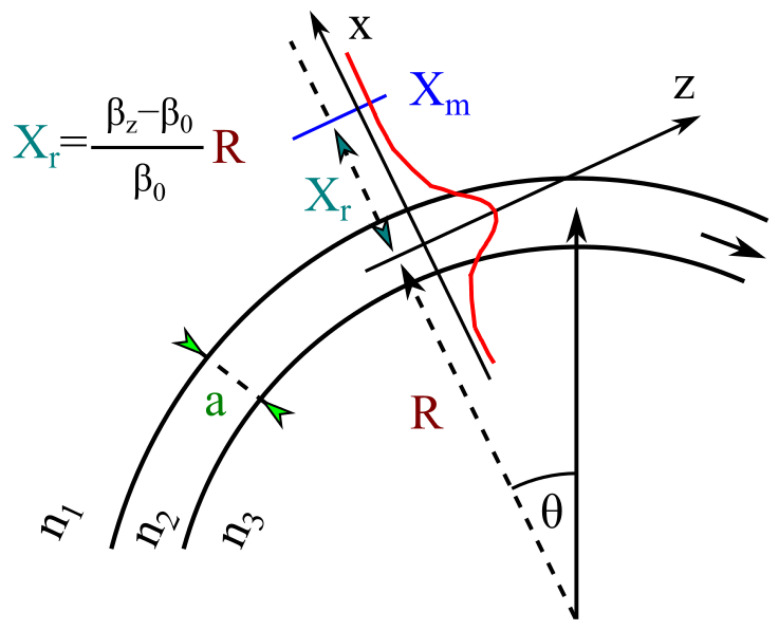
Schematics of the mode in a bent waveguide [27].

We evaluated the bending losses by taking an overlap integral between the straight waveguide mode and its curved counterpart, using the built-in mode overlap functionality in Lumerical MODE. Equation (4) shows the dependence of the radiation loss on the radius of the curvature of the waveguide. Figure 3 schematically shows the propagation of the radiated mode in a bend. It is worth noting that the solution of the wave equation changed with the radius of curvature of the bend, which is shown in Equation (4) for the symmetrical waveguide.

The numerical study focuses on determining the optimal waveguide configuration that balances high confinement factors and minimal bending losses. The analysis comprehensively evaluates different waveguide geometries, considering the trade-offs between the bending radius and larger interaction length in the losses perspective. Fabrication feasibility was also a key consideration, and the analyzed designs were selected to align with standard CMOS photolithographic constraints, ensuring compatibility with scalable, mass-production processes. Although smaller or more optimized dimensions could, in principle, be realized using electron-beam lithography, the present design choices reflect dimensions that are manufacturable using mainstream photolithography.

## 3. Results

### 3.1. Confinement Factor

**Figure 4:**
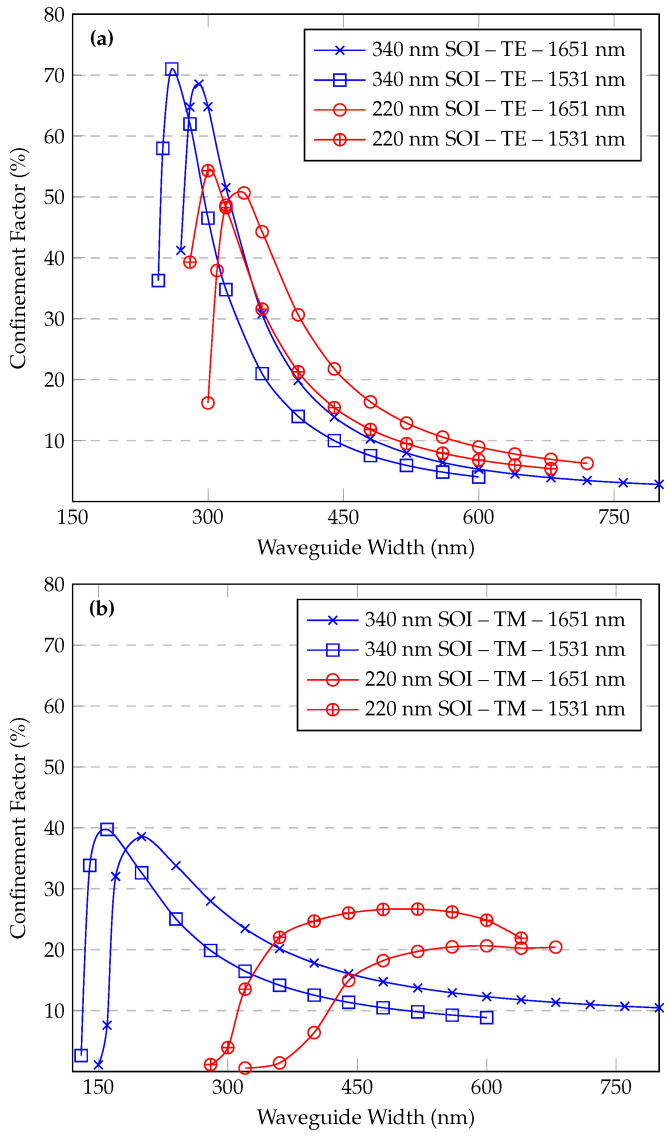
(**a**) TE, (**b**) TM. Confinement factor vs. ridge waveguide width for different wavelengths and SOI heights.

This work focuses on standard silicon-on-insulator (SOI) platforms with silicon heights of 220 nm and 340 nm, which are commonly employed in the silicon waveguide industry. The confinement factor Γ is shown in Figure 4 for both TE and TM modes of a ridge waveguide at wavelengths of 1651 nm and 1531 nm, plotted against the waveguide width. These wavelengths were chosen as representative absorption lines for the greenhouse gases methane and ammonia, respectively.

For the SOI waveguide with a height of 340 nm, the ridge structure supports only the TM mode for widths up to 260 nm. Its width is 270 nm, and it also supports the TE mode. The TE mode achieves its highest confinement factor (Γ) of 68.5% at a width of 290 nm. In contrast, for the 220 nm height, the TE mode shows similar results but with a lower maximum Γ of 50.7%, which occurs at a slightly larger waveguide width of 340 nm. However, for the TM mode, the 220 nm platform presents a different curve with a significantly lower Γ. For widths of 720 nm and above, the components $E_{x}$ and $E_{y}$ of the TM mode correspond to a second-order mode, and a third-order mode begins to be supported at a width of 680 nm.

It is observed that for the TE mode, reducing the height from 340 nm to 220 nm causes only a slight shift in the waveguide width corresponding to the maximum Γ. However, for the TM mode, this change dramatically alters the waveguide’s mode support, preventing the TM mode from being supported as a single mode at smaller waveguide widths.

For the ridge architecture, the TE mode consistently exhibits higher Γ for both height options across a wide range of waveguide widths.

**Figure 5:**
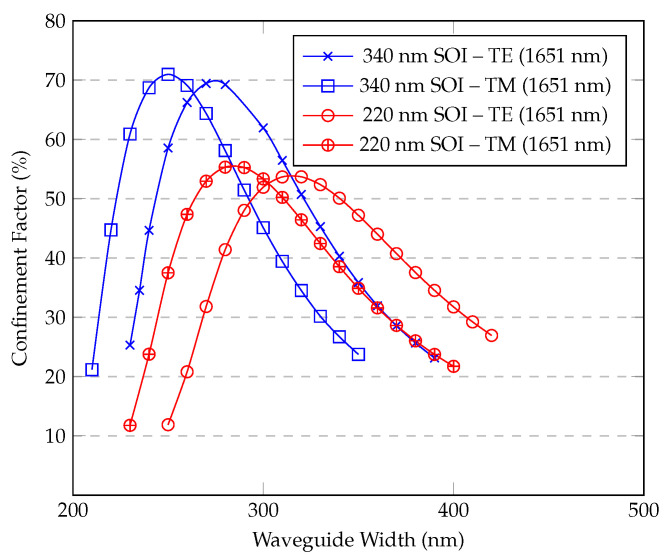
Confinement factor vs. slot waveguide strip width for different wavelengths and SOI heights.

Slot is another typical waveguide architecture used for gas sensing. Figure 5 shows the confinement factor results for the slot waveguide. It is shown as a function of the slot rail width. This figure includes the results for the 1651 and 1531 nm wavelengths together. The slot architecture results are close to the ridge waveguide with approximately 70 % and 50 % for the 340 nm SOI and the 220 nm SOI platforms, respectively.

**Figure 6:**
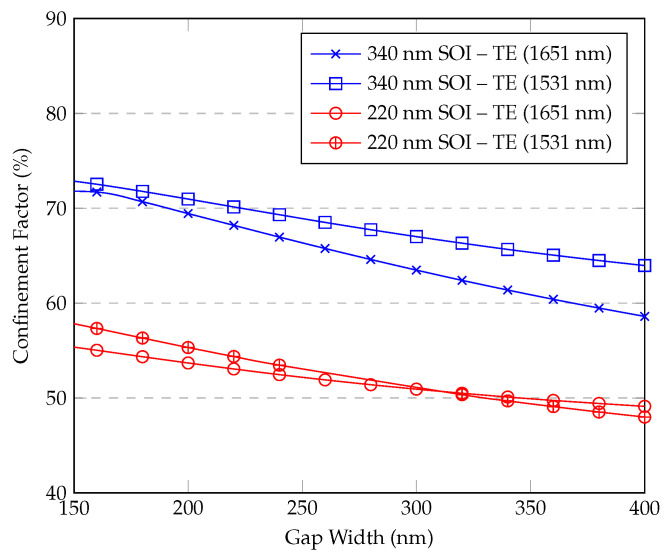
Confinement factor vs. slot waveguide gap width for different wavelengths and SOI heights.

We then analyzed the confinement factor in slot waveguides. Slot waveguide is an optical waveguide that guides light through a subwavelength-scale, low-refractive-index region (the “slot”), which is the gap between two high-refractive-index strips, enabling strong confinement of light in a small area (the gap). Figure 6 shows the variation in the confinement factor with gap width for the fixed parameters of the waveguide, for the highest confinement factor Γ strip width (as per Γ in Figure 5). These results show that the confinement factor decreases with a gap and shows a nearly linear dependency on the gap width. This can be explained by the fact that when the gap between the strips is smaller, the interaction between the two modes within the strips increases. This increased interaction leads to a higher fraction of the intensity within the gap.

Within the range of gap widths investigated, the confinement factor undergoes a measurable change. Specifically, as the gap width increases, the confinement factor tends to decrease, although the magnitude of this reduction is relatively moderate. From an application standpoint, this implies that moderate variations in the slot width will not drastically impact the waveguide’s performance.

Moreover, it can be inferred that the slot waveguide is relatively tolerant to fabrication inaccuracies in the gap dimension. While the reduction in confinement factor remains bounded, the effects of minor errors in the slot gap are much less pronounced than similar inaccuracies in the waveguide’s overall width. Hence, any slight deviations in the slot gap would not significantly diminish the waveguide’s operational efficiency or sensing capabilities.

Additionally, the range of slot gap widths considered here aligns with the practical manufacturing tolerances commonly achieved in large-scale waveguide production processes. Although there are specialized manufacturers capable of fabricating smaller feature sizes, the values explored in this study reflect the most widespread and readily available processes. In many standard photolithographic and etching methods, the lower bound of the examined gap widths is near the typical precision limit. As a result, the findings presented here are highly relevant for mainstream industrial fabrication.

### 3.2. Bending Losses

**Figure 7:**
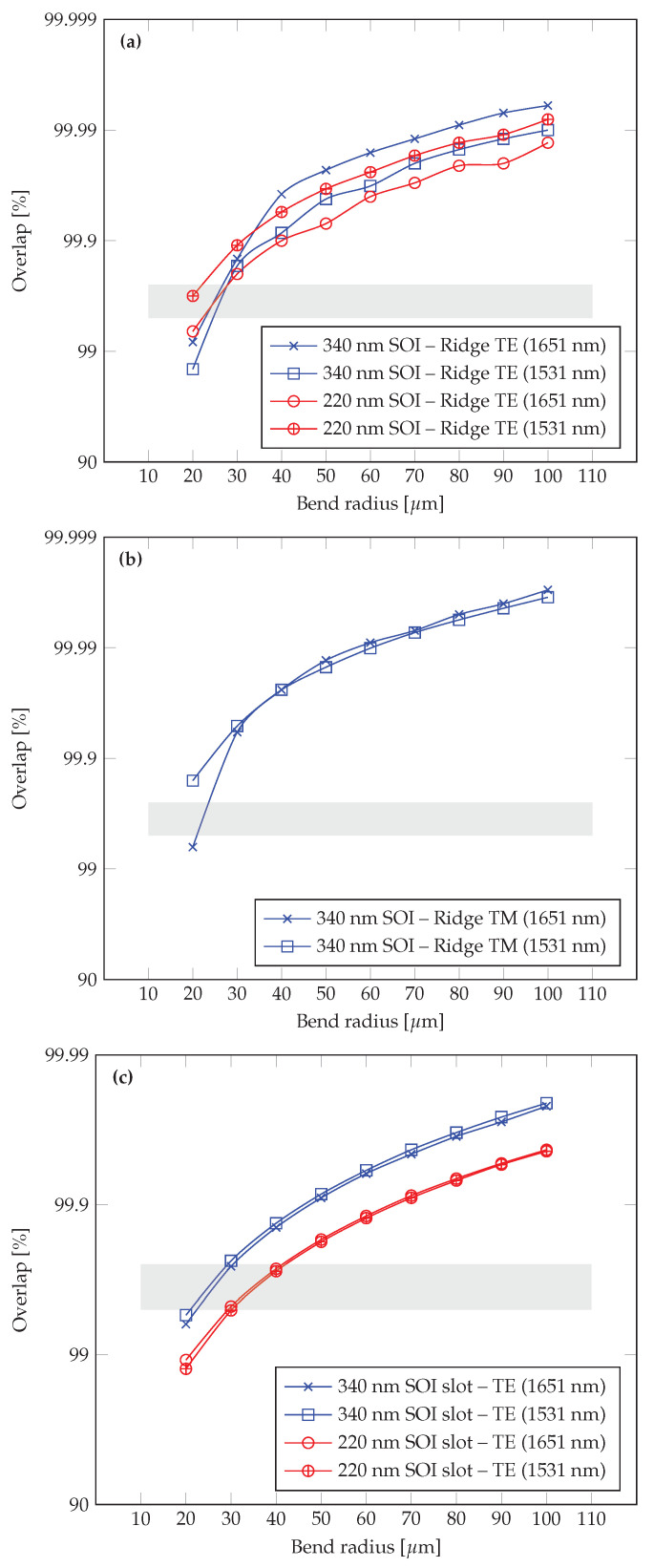
Mode overlap for curved waveguides versus bending radius for different waveguide architectures: (**a**) Ridge TE, (**b**) Ridge TM, (**c**) Slot, and SOI heights. Shaded region indicates the loss range (0.25–0.5%) discussed in the main text as a practical reference for evaluating bending-induced attenuation.

This section presents an additional layer of the analysis, focusing on the influence of the bending radius on the overlap integral in a bent waveguide within the 20–100 μm range. Our overlap simulations were conducted for a single 90° bend, using the dimensions that yielded the highest confinement factor (Γ) for each of the three architectures examined, ridge-TE, ridge-TM, and slot (Figure 4 and Figure 5, excluding the 220 nm SOI ridge-TM waveguide, which was shown to be less suitable for sensing). The results are presented in the accompanying Figure 7 and illustrate how losses change as a function of bend radius.

In practical applications, where optimal chip area utilization is desired, many bends are used to design complex routing paths (e.g., spiral or zigzag layouts). In such cases, it is crucial to account for the fact that overall losses accumulate with each additional bend, following a power law relative to the single-bend overlap. Specifically, if the power transmission for one bend is *O*, then for *N* bends, the total transmission would be $O^{N}$. Moreover, extending the bend to 180° effectively doubles the exponential factor of the losses compared to a 90° bend. Additionally, there is a trade-off between achieving a longer interaction length—crucial for gas-sensing waveguides—and the losses introduced by using smaller bend radii. While a smaller bend radius allows for a longer overall path in a given chip footprint, it also results in higher individual bend losses and an increased number of curves compared to larger bend radii. This underscores the importance of choosing an optimal bend radius to ensure that accumulated losses do not significantly degrade the signal at the waveguide output. For example, on a 1cm chip, using a bend radius of 20μm yields a spacing of approximately 40μm between adjacent waveguide paths (for a “go-and-return” arrangement), allowing about 250 bends to be packed onto the chip. However, each additional bend contributes further losses, so one must balance compactness with acceptable signal attenuation. Indeed, with individual bend losses in the range of 0.25–0.5% per bend, the overall attenuation can reach about 50–75% of the signal after all bends.

From the simulation results, it can be observed that starting at a bend radius of about 50μm and above, the signal transmission remains sufficiently high for standard applications (i.e., the losses are not prohibitively large). Although slot waveguides exhibit slightly higher losses compared to ridge waveguides, even here, only a modest increase in bend radius is required to maintain a usable signal level.

These findings show that a bend radius in the 50μm to 100μm range represents an effective operating point for most applications, balancing the need to fill the chip area with multiple waveguide paths against the requirement to minimize overall bend losses. Nonetheless, slot waveguides—particularly when many bends are involved—may necessitate a slightly larger radius to ensure an adequate signal-to-noise ratio.

Although a figure of merit (FOM = Γ/α) has been suggested for quantitative benchmarking, it is important to note that bending losses (α) strongly depend on the chosen bending radius. Different designs may prioritize interaction length versus optical losses differently, resulting in variations of the bending radius selection. Consequently, reporting a single FOM value might not reflect the full design flexibility and trade-offs inherent in waveguide-based gas sensors. Therefore, instead of a single FOM value, we present detailed parametric results (Figure 4, Figure 5, Figure 6 and Figure 7), allowing designers to clearly evaluate these trade-offs according to their application-specific requirements.

## 4. Discussion

In this work, we performed dual-parameter optimization, addressing both evanescent field confinement (Γ) and bending-induced losses—a combination rarely treated jointly in prior work.

A detailed comparison was performed of ridge and slot waveguides across multiple SOI platform heights (220 nm and 340 nm) and practical near-infrared wavelengths (1531 nm and 1651 nm) relevant to key analytes, such as methane and ammonia.

We have quantified the trade-off between interaction length and curvature-induced attenuation, leading to practical design recommendations, such as optimal bend radii of 50–100 µm. This bend radius range offers a practical balance between minimizing bending losses and preserving chip compactness. For example, within a 1 cm^2^ photonic chip, a bend radius of 50 μm allows for waveguide lengths approaching 1 m, while a 100 μm radius still supports over 0.5 m of cumulative routing length. This enables multiple sensing arms to be integrated compactly, with controlled bending loss.

Insights into fabrication tolerance were reported, demonstrating that slot waveguides offer robust performance despite dimensional variances in slot width, which is crucial for manufacturability.

The study provides CMOS-compatible guidelines for scalable, compact, and high-performance NIR gas sensors, validated through extensive simulations. These characteristics make the proposed designs particularly well-suited for real-time monitoring applications, including methane and ammonia detection, where fast response and high integration density are essential.

To our knowledge, this is the first work to systematically analyze and unify NIR-mode confinement, curvature effects, and manufacturability constraints into a set of design rules for overtone-based photonic gas sensors. For this, we calculated the fundamental eigenmodes of the straight waveguide structure, ensuring an accurate representation of the mode field distributions without curvature. As part of this stage, we examined multiple waveguide architectures and SOI platforms of varying thicknesses, using the mode confinement factor as a key figure of merit for evaluating the suitability of each structure. This metric allowed us to assess the waveguide’s effectiveness for high-efficiency gas detection across different wavelengths, specifically those matching the overtone absorption lines of various gases. Next, we introduced bends into the same waveguide geometry and re-simulated the structure, allowing us to determine how the optical modes were affected by curvature. By computing the overlap integral between the modes of the straight and bent configurations, we quantified the energy transfer and corresponding attenuation associated with bending. This approach enabled a direct, comparative assessment of loss as a function of bend radius and waveguide design parameters, guiding the optimization process toward more efficient, low-loss integrated photonic gas sensors.

By calculating Γ in waveguides for various bend radii, we were able to systematically assess how different curvature values influenced mode confinement and transmission. For each waveguide geometry, we ran multiple simulations, adjusting the bend radius and calculating the corresponding overlap integrals. This comparative analysis allowed us to pinpoint the bend radius that best balanced minimal bending loss with sufficient mode propagation, ensuring that the optical mode remains well-confined while traversing a compact footprint on the chip.

Γ in our calculations is 70%. This is higher than in previous standard waveguide design works, such as Tombez’s work [6], which was instrumental in demonstrating that standard silicon strip waveguides can enable gas sensing in the near-IR, achieving a confinement factor (Γ) of 28.3% for methane detection at 1651 nm. Koompai et al. [16] reached similar Γ values (25–30%) using ${\mathrm{Si}}_{3}$$N_{4}$ slot waveguides around 2 μm, supporting multi-gas detection over a wide spectral range. Higher Γ values have been reported in slow-light structures like photonic crystals and subwavelength gratings [13,14,15], though PhC designs remain more complex to fabricate.

Sensitivity analysis was inherently performed in the parametric simulations (see Figure 4, Figure 5, Figure 6 and Figure 7), clearly showing the dependence of the confinement factor (Γ) on dimensional variations. It was observed that deviations toward smaller dimensions typically had a slightly stronger effect on Γ compared to dimensional increases. With respect to bending losses, further analysis showed that variations within tens of nanometers from the nominal dimensions did not significantly affect performance, confirming that the proposed waveguide designs are robust to realistic fabrication variations typical of standard photolithographic processes.

Importantly, by identifying the optimal bending radius, we can maximize the effective interaction length between the guided light and the target analyte without incurring excessive attenuation. This balance is vital for integrated photonic gas sensors, where maintaining a compact form factor and achieving low loss can significantly improve sensitivity, enable more intricate on-chip layouts, and ultimately lead to more effective and reliable sensing solutions.

These findings are based on numerical modeling and theoretical analyses that align with reported values in the literature, providing a high level of confidence. However, practical factors—such as material heterogeneity, layer arrangement, and fabrication tolerances—may influence final losses. A potential limitation of this approach lies in cross-sensitivity, where multiple gases exhibit overlapping absorption features in the NIR region. Future work may incorporate spectral filtering or multi-wavelength interrogation to improve analyte specificity.

## Data Availability

The data presented in this study are available on request from the corresponding author.
